# Amino acid distribution in blood following high-intensity interval exercise: a preliminary study

**DOI:** 10.1007/s00726-023-03378-y

**Published:** 2024-02-01

**Authors:** Nattai Borges, Thomas M. Doering, Grace Murphy, Margaret Macdonald, Richard H. Dunstan

**Affiliations:** 1 Faculty of Medicine and Health, School of Health Sciences, University of New South Wales, Sydney, Australia; 2 School of Environmental and Life Sciences, University of Newcastle, Newcastle, Australia; 3 School of Health, Medical and Applied Sciences, Central Queensland University, Rockhampton, Australia; 4 InnovAAte Pty Ltd, Newcastle, Australia

**Keywords:** Red Blood Cell, Erythrocyte, Cycle, Plasma, Protein

## Abstract

This study investigated the effect of high-intensity interval exercise on total and individual amino acid concentrations in red blood cells (RBCs) and plasma. Seven males (31 ± 13 yr) provided venous blood samples at rest, immediately and 15 min and 30 min following an 8-min high-intensity exercise bout. The exercise bout was 16 × 15 s cycle efforts at 0.4N/kg of body mass and 90 rpm, interspersed with 15 s passive recovery. Total and individual amino acid concentrations of RBC and plasma and blood cell parameters were analysed. No significant differences for total amino acid concentrations between RBC and plasma were found. Individual amino acid analyses showed significant interaction effects for alanine and α-aminoadipic acid (*P* < 0.05), with plasma alanine significantly increased from baseline across the recovery period (*P* < 0.001). Blood fraction (group) effects showed greater concentrations of glycine, serine, asparagine, aspartic acid, glutamic acid, α-aminoadipic acid and ornithine in RBC, while greater concentrations of alanine, α-aminobutyric acid, valine, leucine, isoleucine, threonine, proline, phenylalanine, glutamine, tryptophan and cystine were found in plasma (*P* < 0.05). Comparable levels of histidine, lysine and tyrosine were observed between blood fractions. Significant differences in the variation of total amino acids in RBC were reported with higher variance at rest compared to following exercise (*P* = 0.01). Haemoglobin, pack cell volume and white blood cell count significantly increased immediately following exercise (*P* < 0.05) but returned to baseline after 15 min recovery. These results support the notion of individualised amino acid transportation roles for RBC and plasma during exercise.

## Introduction

Red blood cells (RBCs) comprise 38–52% of whole blood which facilitates their primary role of gas exchange between the environment and peripheral tissues (Mairbäurl 2013). In contrast, the transport of metabolites, hormones and waste products has been primarily associated as a function of the plasma (Elwyn et al. 1972, 1968; Felig et al. 1973). Although these basic physiological roles for RBC and plasma fractions of blood have long been established, a strong body of evidence supports the hypothesis that RBCs play a pivotal role in the transportation of amino acids (Agli et al. 1998; Divino Filho et al. 1997; Dunstan et al. 2019; MacLaren et al. 2000). Mature RBCs have demonstrated the capacity to synthesise glutathione from glutamic acid, cysteine and glycine for antioxidant protection (Giustarini et al. 2008; Wu et al. 2004). In addition, RBCs also contain seven distinct transporter systems for the uptake of a broad range of proteinogenic amino acids via both facilitated diffusion and active transport (Tunnicliff 1994). Importantly, RBCs do not have a nucleus or mitochondria and generate energy via glycolysis; therefore, RBCs do not undertake protein synthesis nor aerobic respiration (Snyder and Sheafor 1999; Zhang et al. 2011). As a result, there is no metabolic requirement to have high cellular levels of amino acids in RBCs, and yet analyses of RBC cytoplasmic extracts have revealed higher concentrations of certain proteinogenic amino acids than those found in the plasma (Agli et al. 1998; MacLaren et al. 2000). Collectively, these results support the hypothesis that RBCs likely store amino acids for the purpose of transportation.

In support of the hypothesis that RBCs play a transportation role, analyses of RBCs from venous samples have been shown to contain lower total amino acid concentrations than RBCs from arterial samples (Elwyn et al. 1972, 1968; Felig et al. 1973). This arteriovenous difference in RBC amino acid concentration suggests that amino acids are potentially delivered to peripheral tissues and restocked, most likely, via the liver. Previous research in canines has suggested that the plasma may be primarily responsible for returning excess amino acids to the liver where they can be transferred to RBCs for subsequent redistribution (Elwyn et al. 1972). Moreover, a recent study demonstrated that placing RBCs in alternating PBS solutions containing high and low amino acid concentrations could facilitate rapid uptake and release of amino acids (Thorn et al. 2020). It was determined that RBCs could exchange around 15% of their carrying capacity upon exposures to high or low external concentrations of amino acids. Combined, this evidence suggests that RBCs have a functional capacity to take up and release amino acids (Agli et al. 1998; Tunnicliff 1994), which may be consistent with a transportation role for delivery of amino acids in which RBCs are well suited given they are distributed efficiently throughout the whole body. Interestingly, it may be that the plasma and RBCs play differential roles in amino acid distribution throughout the body. Indeed, amino acid distribution is vital in humans as amino acids are involved in molecular signalling pathways and utilised for the biosynthesis of proteins and key metabolites (Wu 2009). In addition, under conditions of extreme stress, certain amino acids are utilised at greater rates and can become conditionally essential (Dunstan et al. 2017; Millward 2004).

Depletion of certain amino acids has been linked to general fatigue and numerous health complications (Wu 2009). Interestingly, resting amino acid levels in RBCs demonstrate large within (MacLaren et al. 2000)- and between-subject variation (Agli et al. 1998; Hagenfeldt and Arvidsson 1980). In addition, concentrations increase following oral ingestion of amino acids (Agli et al. 1998) as well as acutely during and following moderate-intensity exercise (MacLaren et al. 2000). These variations suggest that the transportation capacities of RBCs are likely influenced by multiple parameters and potentially could be optimised via lifestyle interventions to potentially improve health and physical performance outcomes. However pragmatically, little is known about the role of RBCs in amino acid transportation and no studies have investigated the effect of high-intensity exercise on RBC amino acid concentrations. Given the metabolic links between glucose availability and amino acid contributions to metabolism during exercise and recovery (Brooks 1987), a greater understanding of the amino acid profiles within RBCs following high-intensity exercise, where glucose demands are high, could further the current knowledge about the role of RBCs in the transportation of amino acids. Therefore, the aim of this study was to investigate the effect of high-intensity interval exercise on total and individual amino acid concentrations in RBCs and plasma. The authors hypothesised that RBCs and plasma would contain differential profiles of individual amino acids at rest and demonstrate individual responses following high-intensity exercise.

## Methods

### Subjects

Seven healthy males were recruited to participate in this study (age: 31 ± 13 yr, body mass: 83.9 ± 16.2 kg, height: 181.5 ± 6.4 cm). To be eligible for the study, all subjects were required to be free from injury and medication that may have affected their ability to perform exercise. Prior to inclusion, all subjects were informed about the potential risks and benefits of involvement in the study and were required to give written consent. The University of Newcastle Human Ethics Research Panel approved this study.

### Procedures

This study consisted of a single session in which venous blood samples were taken before and throughout 30 min of supine recovery following an 8-min high-intensity interval exercise (cycle) protocol. All venous samples were taken by a trained phlebotomist from the antecubital vein in a supine position. At arrival to the laboratory following an overnight fast, a baseline venous blood sample was taken followed by subsequent samples taken immediately following cessation of exercise, 15 min post-exercise and 30 min post-exercise. Venous blood samples were collected into an EDTA (4 mL) and a lithium heparin (9 mL) vacutainers for subsequent analysis of blood cell parameters and amino acid concentrations of RBC and plasma fractions.

### Exercise protocol

The high-intensity interval exercise protocol was performed on a Monark 894E cycle ergometer (Varberg, Sweden). Following the resting venous blood sample, a 6-min warmup was performed on the cycle ergometer at 100 W with 3 s maximal accelerations at the end of each of 3 min portion of the warmup. The subjects then rested for 3 min while seated on the cycle ergometer before the commencement of the exercise protocol. The exercise protocol was an 8-min bout, consisting of 16 × 15 s efforts interspersed with 15 s passive recovery (W:R = 1:1). The efforts were set to a resistance equivalent to 0.4N/kg of body mass, and the subjects were instructed to cycle at 90 revolutions per min (rpm). During the passive recovery bouts, the subjects were instructed to remain static on the cycle ergometer until the next exercise effort. Upon completion of the exercise protocol, the subjects immediately stepped off the ergometer and lay supine on a plinth adjacent to the ergometer, for a standardised 30 min supine recovery in a quiet room.

### Haematological analysis

The EDTA vacutainers were immediately transported and analysed for standard haematology testing via a third-party NATA-accredited pathology service (Medtech Services Pty Ltd) to provide the blood cell data (RBC count, haemoglobin, packed cells, mean corpuscular volume, mean corpuscular haemoglobin, mean corpuscular haemoglobin concentration, white cells, neutrophils, lymphocytes and monocytes). These data, in particular RBC parameters, are important to provide overall analysis of RBC quantity in circulation. The lithium heparin samples were centrifuged at 2000 rpm for 15 min at 4 °C with the upper plasma layer transferred to an Eppendorf tube for subsequent amino acid analysis. The white cell buffy coat layer was removed, and the remaining RBCs were washed three times in pre-chilled sterile phosphate-buffered saline (PBS). A 200-uL aliquot of the washed RBC was transferred to a fresh Eppendorf tube containing 200 uL Milli-Q H_2_O to lyse the cells. This solution was allowed to settle for 5 min before microfuging at 15,000 × g for 15 min. The lysate supernatant was filtered on QIAgen spin columns by centrifuging at 15,000 × g for 5 min. The filtrate (100 uL) was then transferred to a clean reaction tube containing 200 µL Milli-Q H_2_O with 100 uL 0.2 mM nor-valine in 10% propanol and 20 mM HCl, as the internal standard. The plasma and RBC lysate samples were then prepared for amino acid analyses using a commercially available EZ:Faast^™^ (Phenomenex^®^ Inc.) derivatization kit allowing analyses by gas chromatography with flame ionization detection (GC/FID) to provide individual amino acid concentrations as described previously (Badawy 2019; Dunstan et al. 2015).

### Statistical analyses

All data were presented as mean ± standard deviation (SD). The distribution of all data was tested with the Shapiro–Wilk normality test. The time course for the blood cell parameters was assessed with a separate one-way repeated-measures ANOVA to assess any change in blood cell parameters across the exercise and recovery bout. In addition, the total and individual amino acid concentrations in RBCs and plasma across the exercise and recovery bout were analysed using separate two-way repeated-measures ANOVAs to assess any blood fraction (group)–time interactions. If significant interaction or blood fraction effects were present, data were further analysed using Bonferroni post hoc analyses. Levene’s test of equality was also used to assess differences in variation in the total amino acid concentrations in RBCs and plasma across the four time points. All statistical analyses were conducted using Statistica software package (StatSoft. Inc., Tulsa, USA), and statistical significance was accepted at *P* < 0.05.

## Results

**Fig. 1:**
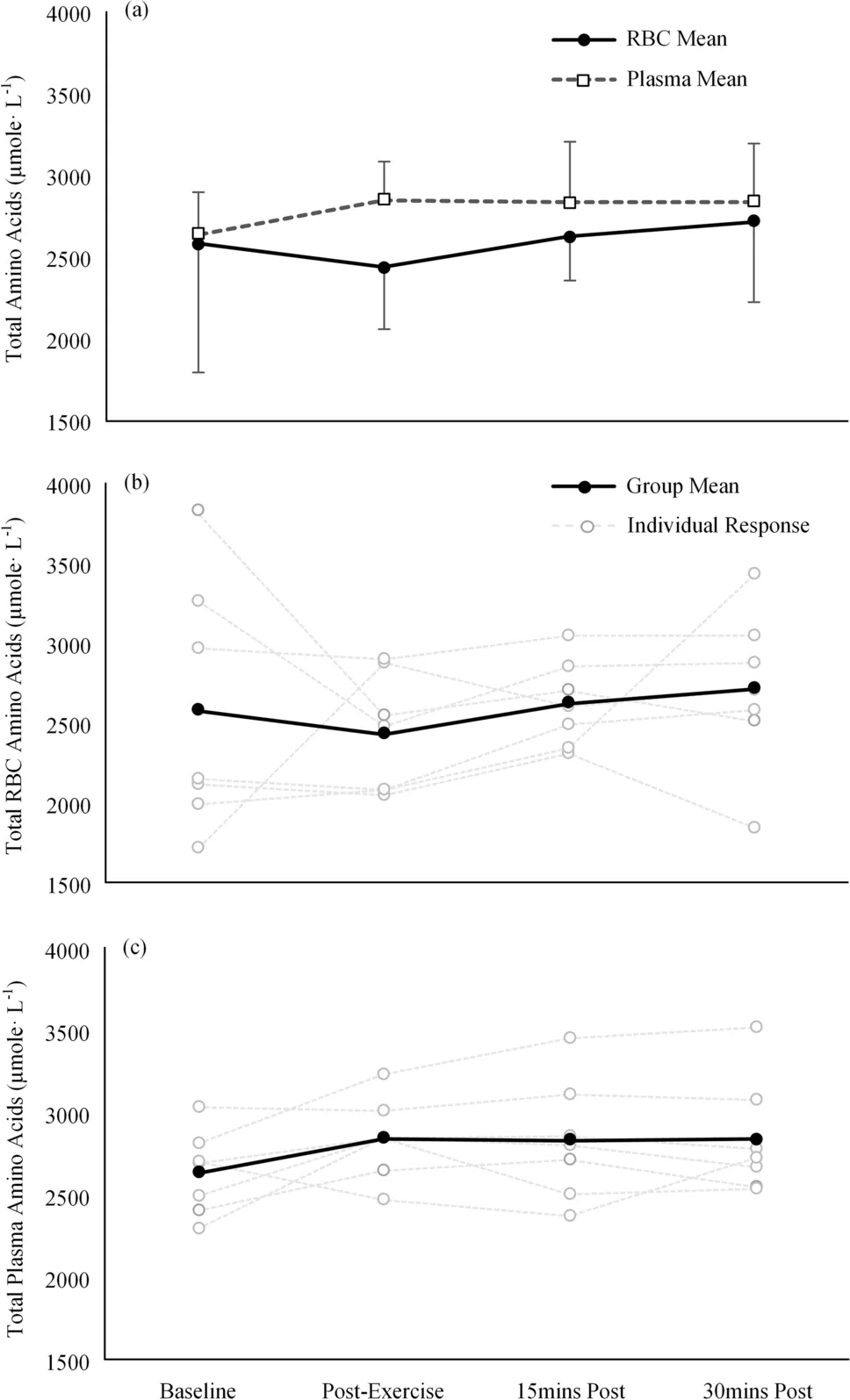
Mean and individual responses for total amino acid concentrations in RBCs and plasma across the exercise and recovery sessions

The mean resistance force and power outputs on the cycle ergometer during the exercise efforts were 33.8 ± 5.9 N and 304.5 ± 53.0 W, respectively. The mean and individual responses for total amino acid concentrations in RBC and plasma across the exercise and recovery bout are presented in Fig. 1. No significant interaction or main effects were found when comparing the total amino acid concentrations in RBC and plasma across the exercise and recovery bout (*P* > 0.05). High levels of between-subject variation were evident in the resting RBC total amino acid concentrations which were seen to reduce following exercise (Fig. 1b). Leven’s test of equality demonstrated a significant difference in the variance for the total amino acid concentrations in RBCs across the four time points (*P* = 0.01) and no significant difference across the plasma time points (*P* > 0.05).

All individual amino acid concentrations in the RBCs and plasma across the exercise and recovery bout are presented in Table 1. Significant interaction effects were found for alanine (*P* = 0.02) and α-aminoadipic acid (*P* = 0.02). Post hoc analyses only demonstrated a significant increase in plasma alanine that remained significantly elevated from baseline levels throughout the entire recovery period (*P* < 0.001). Significant blood fraction effects showed greater concentrations of glycine, serine, asparagine, aspartic acid, glutamic acid, α-aminoadipic acid and ornithine in RBCs. In contrast, greater concentrations of alanine, α-aminobutyric acid, valine, leucine, isoleucine, threonine, proline, phenylalanine, glutamine, tryptophan and cystine were in the plasma (*P* < 0.05). Comparable levels of histidine, lysine and tyrosine were exhibited in RBCs and plasma (*P* > 0.05).

**Table 1:** Mean ± standard deviation of amino acid concentrations (µmole·L^−1^) in red blood cells and plasma across the exercise and recovery sessions

Amino acid	Red blood cells							
	Baseline	Post-exercise	15 min post	30 min post	Baseline	Post-exercise	15 min post	30 min post
Glycine^#^	428.1 ± 111.0^‡^	426.5 ± 99.2^‡^	401.4 ± 66.3	408.3 ± 85.9	267.1 ± 53.2	275.0 ± 49.4	267.7 ± 48.0	267.4 ± 50.4
Aspartic acid^#^	209.5 ± 108.0^‡^	144.7 ± 101.0	241.4 ± 140.4^‡^	216.8 ± 173.3^‡^	2.0 ± 5.2	0.0 ± 0.0	3.3 ± 6.2	0.0 ± 0.0
Glutamic acid^#^	205.5 ± 99.3^‡^	169.1 ± 61.7^‡^	207.1 ± 67.5^‡^	216.2 ± 77.0^‡^	46.4 ± 15.3	49.8 ± 11.5	57.8 ± 18.4	55.7 ± 18.4
Serine^#^	159.2 ± 65.9	149.4 ± 33.2	163.9 ± 23.4	166.5 ± 43.1	118.4 ± 24.2	109.3 ± 26.4	129.0 ± 19.1	119.7 ± 23.4
Ornithine^#^	125.7 ± 125.7^‡^	129.5 ± 33.4^‡^	122.7 ± 30.5^‡^	135.3 ± 36.1^‡^	64.6 ± 21.1	62.4 ± 17.0	60.5 ± 20.3	59.9 ± 18.3
Asparagine^#^	108.5 ± 10.5^‡^	106.2 ± 14.8^‡^	103.1 ± 10.5^‡^	108.3 ± 11.0^‡^	59.1 ± 8.3	58.7 ± 8.2	61.7 ± 10.4	62.0 ± 9.2
α-aminoadipic acid^*#^	13.3 ± 8.0	8.2 ± 7.8	8.0 ± 7.6	16.1 ± 4.3^‡^	4.2 ± 5.9	3.0 ± 4.0	5.8 ± 7.6	3.3 ± 4.6
Glutamine^#^	303.0 ± 170.4	259.0 ± 115.5	314.3 ± 101.9	323.1 ± 107.5	404.0 ± 75.6	416.6 ± 79.4	426.6 ± 82.7	445.4 ± 1.05.7
Alanine^*#§^	281.6 ± 94.5	318.4 ± 35.3	311.0 ± 70.0	318.2 ± 70.4	294.4 ± 51.8	425.0 ± 89.7^†^	424.2 ± 111.3^†^	414.9 ± 98.4^†^
Valine^#^	111.4 ± 59.0^‡^	99.5 ± 36.8^‡^	102.6 ± 27.9^‡^	128.8 ± 77.3^‡^	293.0 ± 44.7	301.5 ± 40.3	284.3 ± 38.2	283.5 ± 43.6
Proline^#^	140.4 ± 39.2	140.7 ± 22.5^‡^	135.4 ± 29.1^‡^	142.5 ± 29.9^‡^	224.1 ± 58.3	247.9 ± 49.7	237.7 ± 62.0	249.3 ± 50.2
Threonine^#^	127.4 ± 53.7	119.6 ± 14.1^‡^	118.6 ± 24.6	125.7 ± 28.1	180.7 ± 35.7	190.9 ± 33.2	183.3 ± 46.2	190.7 43.4
Leucine^#^	51.3 ± 18.3^‡^	51.3 ± 11.3^‡^	57.7 ± 14.2^‡^	54.4 ± 13.0^‡^	134.0 ± 20.2	143.9 ± 19.6	133.1 ± 13.5	129.6 ± 17.8
Isoleucine^#^	14.5 ± 12.5^‡^	9.8 ± 14.8^‡^	12.4 ± 13.6^‡^	10.6 ± 10.2^‡^	65.8 ± 12.5	70.5 ± 12.3	62.2 ± 9.7	61.0 ± 12.4
Phenylalanine^#^	26.9 ± 10.5^‡^	26.4 ± 8.9^‡^	30.7 ± 8.3^‡^	28.8 ± 6.5^‡^	63.6 ± 10.4	66.7 ± 10.8	67.8 ± 9.4	65.8 ± 9.7
Tryptophan^#^	8.9 ± 5.4^‡^	9.2 ± 5.1^‡^	10.8 ± 2.2^‡^	11.4 ± 3.0^‡^	53.4 ± 12.5	49.4 ± 12.9	50.9 ± 14.0	50.1 ± 13.9
Cystine^#^	0.0 ± 0.0^‡^	0.0 ± 0.0^‡^	0.0 ± 0.0^‡^	0.0 ± 0.0^‡^	29.4 ± 12.6	32.0 ± 15.2	33.1 ± 17.2	± 17.7
Methionine^#^	7.0 ± 7.1^‡^	6.4 ± 6.7^‡^	9.0 ± 5.8^‡^	8.8 ± 5.3^‡^	22.2 ± 3.1	25.6 ± 4.1	25.8 ± 3.6	24.0 ± 3.7
α-aminobutyric acid^#^	8.2 ± 7.8^‡^	10.9 ± 5.0^‡^	8.8 ± 6.4^‡^	8.3 ± 5.9^‡^	19.9 ± 4.1	20.5 ± 3.9	18.6 ± 4.3	19.7 ± 4.7
Hydroxyproline^#^	0.0 ± 0.0^‡^	0.0 ± 0.0^‡^	0.0 ± 0.0^‡^	0.0 ± 0.0^‡^	16.8 ± 5.3	15.5 ± 4.2	16.1 ± 4.4	16.8 ± 3.4
Lysine	119.7 ± 34.7	125.4 ± 29.1	125.5 ± 19.1	140.4 ± 38.9	149.9 ± 29.9	155.2 ± 37.2	152.0 ± 39.1	160.7 ± 37.3
Histidine	77.3 ± 28.6	69.7 ± 22.1	69.4 ± 14.5	79.8 ± 21.9	64.3 ± 10.5	64.0 ± 16.5	69.5 ± 19.8	65.6 ± 18.2
Tyrosine	49.1 ± 16.1	49.4 ± 20.1	49.2 ± 12.9	57.8 ± 18.0	57.8 ± 11.6	60.7 ± 12.9	59.6 ± 17.5	58.6 ± 15.1

Significance accepted at P < 0.05 level. Amino acid presentation order is based on blood fraction effects, i.e., RBC, then plasma, then no effect

* = Significant interaction effect, # = Significant blood fraction effect, § = Significant time effect, † = Significantly different from baseline value, ‡ = Significantly different between red blood cells and plasma

**Fig. 2:**
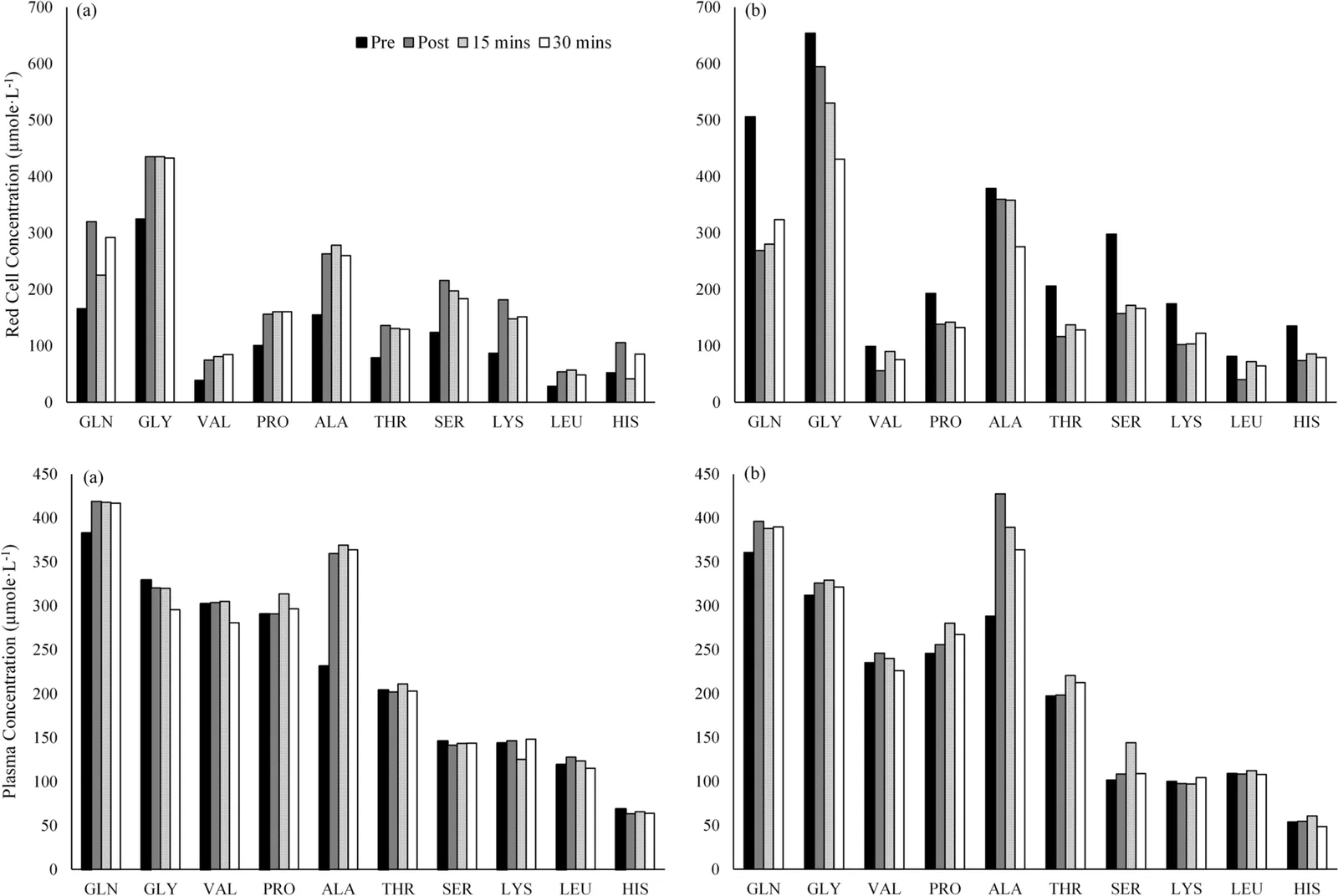
Comparison of individual red blood cell and plasma amino acid concentrations for two respective subjects [(a) vs (b)] across the exercise and recovery sessions. Subject (**a**) had a below-group average red blood cell total amino acid concentration at rest, while subject (**b**) had an above-group average red blood cell total amino acid concentration. *GLN* = Glutamine, *GLY* = Glycine, *VAL* = Valine, *PRO* = Proline, *ALA* = Alanine, *THR* = Threonine, *SER* = Serine, *LYS* = Lysine, *LEU* = Leucine, *HIS* = Histidine

Similar to the total amino acid trends, Fig. 2 highlights the variability in RBC concentrations at an individual amino acid level by presenting plasma and RBC individual amino acid concentrations for two respective subjects [(a) vs (b)] across the exercise and recovery bout. These subjects were selected based on their inversed resting RBC amino acid concentrations (below- vs. above-group average) and demonstrate the differences in resting concentrations and the inverse response to exercise in the RBC amino acid concentrations. Immediately following exercise, the RBC amino acid concentrations between these two representative subjects became much more uniform and seem more tightly regulated following exercise.

**Fig. 3:**
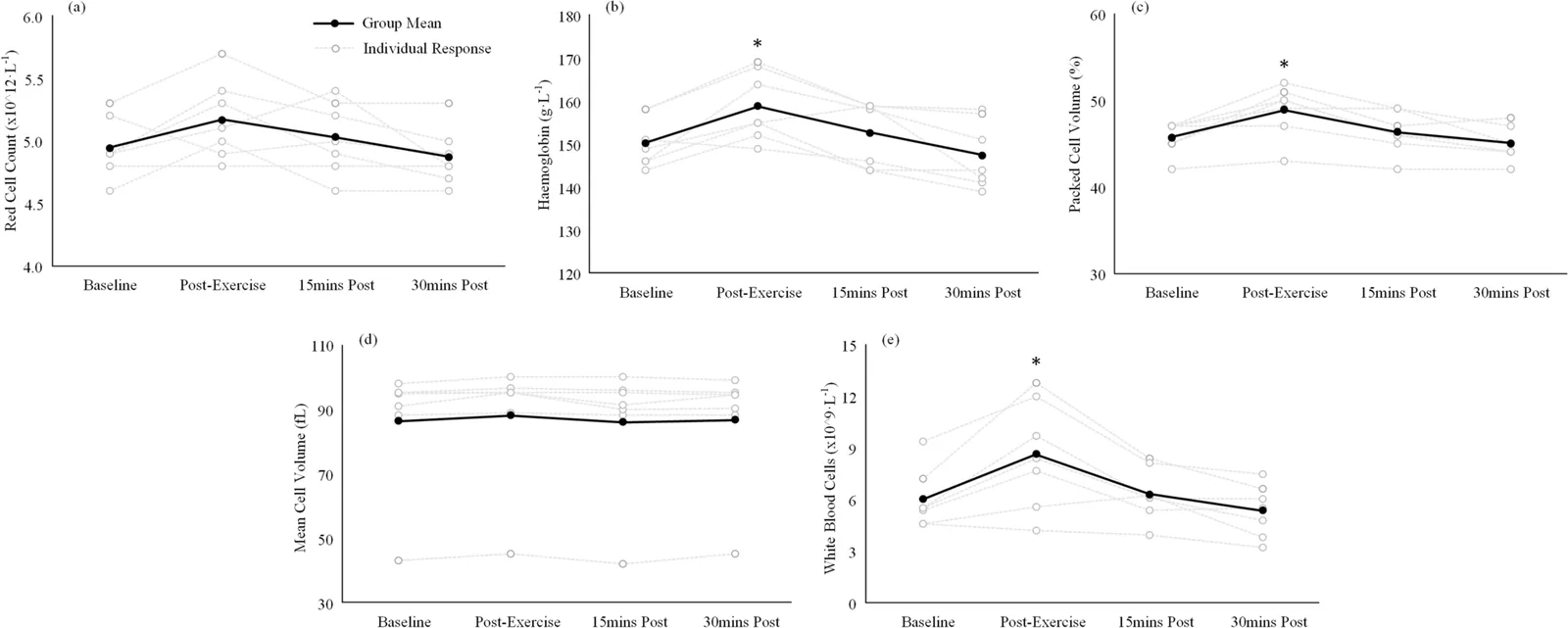
Blood cell parameters that demonstrated significant change across the exercise and recovery sessions. * = Significantly different to baseline value

Analysis of blood cell parameters showed significant change across the session for RBC count (*P* = 0.02; pre-post exercise %∆ =  + 4.6%), haemoglobin (*P* = 0.0002; pre-post exercise %∆ =  + 5.7%), pack cell volume (*P* = 0.0002; pre-post exercise %∆ =  + 6.9%), mean cell volume (*P* = 0.02; pre-post exercise %∆ =  + 1.9%) and white blood cell count (*P* = 0.0001; pre-post exercise %∆ = 43.1%) (Fig. 3). However, post hoc analyses only revealed a significant increase in haemoglobin, pack cell volume and white blood cell count immediately post-exercise compared to baseline, 15 min and 30 min post-exercise (*P* < 0.05). The exercise protocol had no effect on baseline values for corpuscular haemoglobin (28.3 ± 6.1 pg), mean corpuscular haemoglobin concentration (329.7 ± 16.7 g∙L^−1^), neutrophils (5.0 ± 2.1 × 10^9^∙L^−1^), lymphocytes (2.3 ± 1.2 × 10^9^∙L^−1^) and monocytes (0.3 ± 0.1 × 10^9^∙L^−1^) (*P* > 0.05).

## Discussion

The aim of the present study was to investigate the effect of high-intensity interval exercise on total and individual amino acid concentrations in RBCs and plasma. The main findings of this study were: (i) total amino acid concentrations in RBCs and plasma were comparable at all time points; (ii) high levels of between-subject variation were evident for total and individual amino acid concentrations in resting RBCs, with variability reducing immediately post-exercise; (iii) plasma alanine was the only amino acid that demonstrated an interaction effect with significant post hoc results; (iv) differential profiles of individual amino acids were apparent in RBCs and plasma with significant blood fraction effects and (v) significant changes in red blood parameters such as red cell count, haemoglobin and packed cell volume were evident across the exercise and recovery bout. Collectively, these results support the notion that RBCs and plasma may play individualised transportation roles for amino acids during exercise.

The finding of comparable total amino acid concentrations in RBCs and plasma is supported by previous research and supports the notion that further investigation into the role that RBC may play in the transportation of amino acids is warranted (Agli et al. 1998; MacLaren et al. 2000). Although the high-intensity exercise protocol was not a sufficient stimulus to significantly shift total amino acid concentrations between blood fractions in the venous blood, there may be a trend for an inverse pre-post exercise change in amino acid concentrations between blood fractions. Specifically, the amino acid concentration in total RBCs dropped (%∆ =  − 5.6%; Cohen’s *d* = 0.24) and rose in the plasma (%∆ = 7.9%; Cohen’s *d* = 0.84) in the venous blood following the exercise bout. Although not statistically significant, the is inverse response trend supports suggestions from early animal models that RBCs and plasma may play individualised transportation roles for amino acids in the blood (Elwyn et al. 1972).

Inspection of the individual responses for the total amino acids in RBCs and plasma illustrates a large between-subject variation in resting RBC total amino acids, suggesting highly individualised resting RBC amino acid status (Fig. 2b). Despite a standardised overnight fast, this between-subject variation suggests that resting RBC amino acid concentrations are likely influenced by lifestyle and other factors. However, without further lifestyle information, the underlying contributing factors to these large resting variations in RBC amino acids remain difficult to elucidate. Interestingly, upon completion of the cycle protocol, the between-subject variation in total RBC amino acids decreased, suggesting a potential greater requirement to regulate RBC amino acids during exercise. This change in variance was not witnessed in the plasma amino acid concentrations which remained relatively consistent between subjects. The increased regulation of RBC amino acids is also evident at the individual amino acid level for ornithine, aspartic acid, glutamic acid, alanine and glutamine, all of which demonstrated a marked drop in group variance following exercise. This reduced variation of specific amino acids in RBCs suggests a potential specific requirement for these amino acids in response to exercise. Therefore, further studies should investigate whether targeted exercise or nutritional interventions further influence the transportation of these individual amino acids in RBCs in an aim to improve metabolic health and physical performance.

Despite the comparable total amino acid concentrations between RBCs and plasma, the individual amino acid concentrations demonstrated significant interaction effects for alanine and α-aminoadipic acid. This suggests that these amino acids had independent RBC and plasma responses to the cycle protocol; however, the α-aminoadipic acid concentrations were relatively low for both RBCs and plasma, making the pragmatic effect of this unclear. Alanine concentrations increased in both RBCs (%∆ = 13%, Cohen’s *d* = 0.52) and plasma (pre-post exercise %∆ = 45%, Cohen’s *d* = 1.53) immediately following exercise, but plasma alanine was the only amino acid that significantly increased and remained at elevated concentration across the session. This increase in plasma alanine is likely related to the glucose–alanine cycle in which alanine is directed to the liver to produce glucose (Felig 1973; Felig and Wahren 1971). Again, this supports previous suggestions that plasma may be directing amino acids to the liver, while RBCs are being restocked with amino acids at the liver to deliver to peripheral tissues (Elwyn et al. 1972). Previous research has also demonstrated significant increases in plasma alanine across a 90-min moderate-intensity cycle session (65% VO_2max_) (MacLaren et al. 2000). However, the authors also found significant changes in plasma tyrosine, valine, leucine and isoleucine as well as all RBC amino acids during the cycle exercise protocol and into recovery (MacLaren et al. 2000). These differing results are likely contributed to by differences in the exercise protocols given that longer-duration continuous exercise may have a more pronounced effect due to the greater flux in metabolic processes involving amino acids such as protein oxidation, gluconeogenesis and ketone formation (Brooks 1987).

Blood fraction comparisons of individual amino acid concentrations between RBCs and plasma demonstrated greater concentrations of 7/23 amino acids in the RBC, 13/23 in the plasma and comparable concentrations for 3/23 amino acids. This result compared with that of previous research suggests that a greater or similar number of individual amino acids are present in greater concentrations in RBCs compared to the plasma (Agli et al. 1998; MacLaren et al. 2000). In fact, MacLaren et al. (2000) reported that all amino acid concentrations were greater in RBCs compared to the plasma with the exception of arginine and tryptophan. These differing between-study results in the individual amino acid profiles could be related to differences in the haematological analysis (Agli et al. 1998), dietary intake and physical fitness levels of subjects (MacLaren et al. 2000). In addition, between-study differences in the individual amino acid concentrations could also be a manifestation of the demonstrated large between-subject variability in resting RBC amino acid concentrations which is supported by a number of other studies (Aoki et al. 1973; Hagenfeldt and Arvidsson 1980; MacLaren et al. 2000). Given the different outcomes regarding individual amino acid concentrations in RBCs at baseline and in response to exercise, more investigation is warranted to further understand what influences the amino acid concentration values in RBCs.

The significant changes witnessed in the blood cell parameters across the session could also further influence the circulating RBC amino acid levels. Significant main effects for time were observed for red cell count, haemoglobin, pack cell volume, mean cell volume and white blood cell count with significant increases from baseline in haemoglobin, pack cell volume and white blood cells. The changes in red cell parameters may be related to shifts in extracellular fluid or a surplus in RBCs originating from the spleen, which has been suggested to act as a RBC reservoir at rest (Stewart and McKenzie 2002). In fact during exercise, the spleen has been suggested to facilitate the 50% increase in circulating RBCs in horses (Thomas and Fregin 1981) and the 4–5% increase in haematocrit (pack cell volume) in humans (Laub et al. 1993). The previously published 4–5% increase in haematocrit in humans following exercise (Laub et al. 1993) is in line with the data from the present study demonstrating a 4.6%, 5.7% and 6.9% increases in red cell count, haemoglobin concentration and pack cell volume, respectively. Although the authors postulate that the increase in red cell parameters is likely due to an increased requirement for oxygen, it is also logical to assume this would also offer further capacity for RBCs to also transport free amino acids.

## Conclusions

Total amino acid levels in both RBC and plasma are comparable at rest and following high-intensity cycle exercise. High between-subject variance is evident in resting total and individual RBC amino acids, which seems to reduce immediately following exercise, suggesting tighter regulation of RBC amino acids. Differential individual amino acid profiles between RBCs and plasma and an interaction effect for alanine support the notion of individualised transportation roles of amino acids for RBCs and plasma. Future research should explore what potential lifestyle or disease factors could be contributing to the variance in resting amino acid concentrations in RBCs. In addition, further investigations to explain how differential amino acid profiles in blood fractions facilitate and support exercise is warranted.

## Data Availability

Data can be made available on request.
